# Genome-Wide Association of New-Onset Hypertension According to Renin Concentration: The Korean Genome and Epidemiology Cohort Study

**DOI:** 10.3390/jcdd9040104

**Published:** 2022-03-30

**Authors:** Sung-Bum Lee, Byoungjin Park, Kyung-Won Hong, Dong-Hyuk Jung

**Affiliations:** 1 Severance Check-up, Yonsei University Health System, Yongin-si 16995, Korea; dolsoui@yuhs.ac; 2 Department of Medicine, Graduate School, Yonsei University Wonju College of Medicine, Wonju-si 26426, Korea; 3 Department of Family Medicine, Yongin Severance Hosptal, Yongin-si 16995, Korea; bjpark96@yuhs.ac; 4 Healthcare R&D Division, Theragen Bio Co., Ltd., Ganggyo-ro 145, Suwon-si 16229, Korea; 5 Department of Family Medicine, Yonsei University College of Medicine, Seoul 03722, Korea

**Keywords:** hypertension, renin, genome-wide association study, cohort study, Koreans

## Abstract

The renin-angiotensin system (RAS) is a crucial regulator of vascular resistance and blood volume in the body. This study aimed to examine the genetic predisposition of the plasma renin concentration influencing future hypertension incidence. Based on the Korean Genome and Epidemiology Cohort dataset, 5211 normotensive individuals at enrollment were observed over 12 years, categorized into the low-renin and high-renin groups. We conducted genome-wide association studies for the total, low-renin, and high-renin groups. Among the significant SNPs, the lead SNPs of each locus were focused on for further interpretation. The effect of genotypes was determined by logistic regression analysis between controls and new-onset hypertension, after adjusting for potential confounding variables. During a mean follow-up period of 7.6 years, 1704 participants (32.7%) developed hypertension. The low-renin group showed more incidence rates of new-onset hypertension (35.3%) than the high-renin group (26.5%). Among 153 SNPs in renin-related gene regions, two SNPs (rs11726091 and rs8137145) showed an association in the high-renin group, four SNPs (rs17038966, rs145286444, rs2118663, and rs12336898) in the low-renin group, and three SNPs (rs1938859, rs7968218, and rs117246401) in the total population. Most significantly, the low-renin SNP rs12336898 in the *SPTAN1* gene, closely related to vascular wall remodeling, was associated with the development of hypertension (*p*-value = 1.3 × 10^−6^). We found the candidate genetic polymorphisms according to blood renin concentration. Our results might be a valuable indicator for hypertension risk prediction and preventive measure, considering renin concentration with genetic susceptibility.

## 1. Introduction

Hypertension, the primary cause of cardiovascular disease and all-cause mortality, is a leading disease worldwide [1]. Previous estimates suggest that 31.1% (1.39 billion) of adults worldwide had hypertension in 2010 [2]. The prevalence of hypertension has increased globally due to the aging population, exposure to unhealthy foods (i.e., low potassium and high sodium intake), and lack of physical activity. In Korea, hypertension in 2017 was prevalent in 27% of adults over 30 and 60% of adults over 60 [3].

Renin, part of the renin-angiotensin system (RAS), is an enzyme from the kidney’s juxtaglomerular cells, and its secretion mainly responds to renal perfusion pressure [4,5]. Renin hydrolyzes angiotensinogen secreted from the liver into angiotensin I, while circulating through the blood stream. Angiotensin I is converted into angiotensin II by the angiotensin-converting enzyme (ACE). Finally, as a central effector molecule of the RAS, angiotensin II stimulates the release of catecholamines in the adrenal medulla and constricts vessels, resulting in increased blood pressure [6].

RAS activity is often increased in hypertensive patients, and low-renin hypertension has been reported to be around 20–30% [7]. However, epidemiological studies have shown that the degree of RAS dependence on hypertension varies, depending on ethnicity or salt intake [8], which has raised the possibility that genetic susceptibility is one of the underlying mechanisms. Thus, we investigated the genetic predisposition of the blood renin concentration, influencing future hypertension incidence, using a community-based Korean cohort, observed over 12 years.

## 2. Materials and Methods

### 2.1. Study Population and Participants

This study used data from the Korean Genome and Epidemiology Study (KoGES) conducted by the National Research Institute of Health (NIH), Centers for Disease Control and Prevention, Ministry of Health and Welfare, Republic of Korea. The KoGES aims to investigate the genetic and environmental etiologies of non-communicable diseases, such as hypertension and type 2 diabetes. A total of 10,030 participants between the ages of 40 and 69 was recruited through two population-based prospective cohort studies conducted in the Ansung (*n* = 5018) and Ansan (*n* = 5012) regions of South Korea. Of the 10,030 participants, genotype data were available for 8840. After excluding hypertensive patients at enrollment, we included 5211 normotensive individuals in the final analysis (2440 men and 2771 women). The KoGES is ongoing, with biannual repeated surveys beginning in 2001–2002. Data from the baseline study to the fifth examination, in 2011–2012, were used in the current study. Detailed information about the KoGES is described in a previous report [9]. The dataset used in this study (Ansan–Ansung cohort) was provided after reviewing and evaluating the Korea Centers for Disease Control and Prevention (http://www.cdc.go.kr/CDC/eng/main.jsp (accessed on 14 March 2022)).

### 2.2. Data Collection

Baseline and follow-up assessment and recruitment were conducted after obtaining informed consent from all participants. Trained physicians examined study subjects according to a standard protocol [9]. Blood pressure was measured in a sitting position with arms supported at the heart level using mercury sphygmomanometers after at least 5 min of rest in a quiet room (Baumanometer, Baum Co. Inc., Copiague, NY, USA). Systolic blood pressure and diastolic blood pressure were defined as the average of both arm readings. Smoking status was determined based on self-questionnaires and classified into two categories: non-smokers (<100 cigarettes in their lifetime) and current smokers (≥100 cigarettes in their lifetime or currently smoking). Blood samples were collected after fasting overnight or for more than 12 h. Serum concentrations of fasting plasma glucose, total cholesterol, triglycerides, and high-density lipoprotein (HDL) cholesterol were measured with an autoanalyzer (Advia 1650, Siemens, Tarrytown, NY, USA). Plasma renin concentration was measured by using Gamma Counter Cobra in a supine position after more than 15 min between 8 and 10 a.m. (Packard, Meriden, CT, USA) [10]. Furthermore, we subdivided renin concentration into low- or high-renin groups based on 3.3 ng/mL, which is the highest tertile value of plasma renin concentration [11] We used the World Health Organization (WHO) diagnostic criteria for type 2 diabetes mellitus: 8-h fasting plasma glucose ≥ 126 mg/dL or HbA1c ≥ 6.5% [12]. In addition, participants who stated that they were receiving hypoglycaemic agents were included in the diabetic group. Newly developed and baseline hypertension were defined as Systolic blood pressure ≥ 140 mmHg, diastolic blood pressure ≥ 90 mmHg, or current use of antihypertensive medication [13].

### 2.3. Genotype Analysis

Genotypes were obtained from the National Institutes of Health (NIH), and the details of the study design have been described previously [14]. Single nucleotide polymorphism (SNP) genotypes of subjects were extracted from the Affymetrix 5.0 SNP microarray. SNPs with missing genotype call rate > 0.1, minor allele frequency < 0.01, or Hardy–Weinberg equilibrium (HWE) *p*-value < 1 × 10^−6^ were excluded. Finally, 333,651 experimentally determined SNPs and 1,000,000 SNPs imputed based on the 100 genome haplotype phase of the Asian panel or the Korean HapMap were used in this study [15].

### 2.4. Statistical Analysis

We conducted genome-wide association studies (GWAS) for the total, low-renin, and high-renin groups. Among 153 SNPs in renin-related gene regions, the effect of genotype was determined by logistic regression analysis between incident hypertension cases and controls after adjusting for confounding variables. The effect of genotype was determined by logistic regression analysis between controls and new-onset hypertension. The thresholds for association criteria were defined as the genome-wide significant level (*p*-value < 5 × 10^−8^) and the genome-wide suggestive level (*p*-value < 1 × 10^−5^).

## 3. Results

### 3.1. Baseline Characteristics of the Study Population

Table 1 shows the characteristics of the study population. The mean follow-up period was 7.6 years, and a total of 1704 individuals (32.7%, 1704/5211) were diagnosed with hypertension. The mean age was 49.1 ± 7.9 years in the normotensive group and 53.1 ± 8.8 years in the new-onset hypertension group (*p*-value < 0.001). We divided the sample by renin level into two groups, a high-renin group and a low-renin group, and the low-renin group showed more incidence rates of new-onset hypertension (35.3%) than the high-renin group (26.5%). Body mass index, fasting plasma glucose, and triglycerides were significantly higher in the new-onset hypertension group. HDL cholesterol and type 2 diabetes were lower and less frequent in the new-onset hypertension group. However, total cholesterol, systolic blood pressure, diastolic blood pressure, and current smoker status were not significantly different between groups. The incidence rates of hypertension between males and females were similar, 0.34 and 0.32, respectively.

### 3.2. Association between SNPs and New-Onset Hypertension

Performing the case-control analysis, we found a significant association between SNPs and new-onset hypertension cases, as listed in Supplementary Table S1. A total of 153 SNPs in renin-related gene regions showed association in all three GWAS. Furthermore, we selected a cluster pick region to represent the more reliable association and summarized the top SNPs of that region in Table 2. An additional column reveals associations by group (L: low-renin group; H: high-renin group; M: both groups). The next column outlines the top significant SNPs among the associated loci, and the final column shows the cluster picked in each locus, with at least three SNPs associated with hypertension with *p*-value < 1 × 10^−5^. Among 153 SNPs in renin-related gene regions, two SNPs (rs11726091 and rs8137145) showed an association in the high-renin group, four SNPs (rs17038966, rs145286444, rs2118663, and rs12336898) in the low-renin group, and three SNPs (rs1938859, rs7968218, and rs117246401) in the total population. Among these SNPs, rs12336898 showed the most significant *p*-value and is located in the 3′ downstream region.

### 3.3. In Silico Annotation of Linked Genes and Functional Relevance

In silico annotation of the positions of SNPs in gene regions is described in Table 3. Five SNPs were located near the functional gene regions, and four were located in intergenic regions. The most significant SNP, rs12336898, was located downstream of the *SPTAN1* gene in the L group. Interestingly, *SPTAN1* (αII spectrin) is one of two α spectrin genes, and its activation is associated with the compensation of vessel fibrosis. In silico annotation of the SNP function showed that rs1938859 is located in the 5′ untranslated region of the *TRPC6* gene and was reported as an expression quantitative trait locus (eQTL) of the same gene, which is related to glomerulosclerosis [16]. Among the SNPs, rs11726091 is associated with the artery and aorta and is an eQTL of the *MSX1* gene.

## 4. Discussion

This large-scale cohort study, over 12 years, found that hypertension occurred more in the low-renin group, among community-dwelling Koreans, and the genetic predisposition influenced the future hypertension incidence, according to renin concentration. Koreans have significantly higher salt intake than Westerners, and the frequency of low-renin hypertension may be higher than commonly known [7,42,43]. In a previous four-year follow-up study conducted in Korea, the low-renin group had a higher incidence of hypertension than the high-renin group, which was noticeable in the individuals with high salt intake [8]. Furthermore, it suggests that different genetic predispositions may affect the occurrence of hypertension, depending on the blood renin concentration.

In this study, there are two SNPs (rs11726091 and rs8137145) in regions associated with high renin level and hypertension. The first SNP (rs11726091) showed a *p*-value of 4.2 × 10^−6^, suggesting an association with increased hypertension incidence. The SNP was located on chromosome region 4p16.2, and there were no functional genes within ±50 kbp, with the nearest genes being *MSX1* and *CYTL1*. The rs11726091 SNP region was associated with body mass index, lung function, and auto-immune diseases [17,18,19]. The eQTL was located in the *MSX1* gene, expressed in artery and muscle tissues (GETX Portal), an indispensable homeodomain transcription factor for cardiac valve morphogenesis [44]. During embryogenesis, the encoded protein functions as a transcriptional repressor through interactions with the core transcription complex components and other homeoproteins. It also plays roles in the limb-pattern formation, craniofacial development, particularly odontogenesis, and tumor growth inhibition. Mutations in this gene, which was once known as homeobox 7, have been associated with non-syndromic cleft lip, with or without cleft palate, Witkop syndrome, Wolf–Hirschhorn syndrome, and autosomal dominant hypodontia [45,46,47,48]. The second SNP (rs8137145) showed a *p*-value of 6.4 × 10^−6^, suggesting an association with protection against hypertension. The SNP is located on chromosome 22q11.23, in an intron of the *SGSM1* gene, which encodes small G protein signaling modulator 1. Diseases associated with *SGSM1* include autosomal dominant adult-onset proximal spinal muscular atrophy and spinal muscular atrophy, with lower extremity predominance [49].

Interestingly, the most significant SNP was rs12336898 (*p*-value = 1.3 × 10^−6^), suggestive of the location of the incidence of hypertension in the low-renin group. The SNP was located on chromosome region 9q34.11, associated with the *SPTAN1* gene. This study identifies a relationship between rs12336898 in the *SPTAN1* gene and hypertension in low-renin subjects. Moreover, our results indicate that polymorphisms in the downstream region of the *SPTAN1* gene contribute to the incidence of hypertension. Pathogenesis of the *SPTAN1* gene and hypertension via the RAS support these findings.

Angiotensin activates calpains, which are calcium-activated proteases, associated with cardiovascular remodeling and inflammatory kidney diseases [50]. These proteins promote fibrosis through several types of pathogenesis. Calpains degrade IκBα in a specific sequence, leading to NF-κB activation and pro-inflammatory cytokines production [51]. They limit glucocorticoids by degrading HSP-90, reducing anti-inflammatory activity [52,53]. They also promote interleukin-1α maturation and activation and induce the onset of inflammatory processes and fibrosis, in the renal and cardiovascular systems [54,55]. Moreover, through AT-1 receptor signaling, angiotensin II induces vascular aging [56]. Therefore, angiotensin can promote vascular and kidney aging because of the breakdown products of spectrin in the kidney cortex and cardiomyocytes [57].

Spectrins play an essential role between the actin-based cytoskeleton and membrane and maintain the cellular structure [56,58]. αII spectrin (*SPTAN1*) is one of two α spectrin genes. Dysfunction of this gene is associated with alterations in axon initial segment formation and cortical lamination. II spectrin-deficient mice present significant structural and cellular phenotypes that result in structural remodeling and fibrosis [59]. If sodium uptake increases, the blood volume expands, and preload increases. Increased preload can result in vessel injury and cardiac remodeling. Therefore, the RAS is down-regulated, and the *SPTAN1* gene is activated for compensation for vessel fibrosis [60].

Building upon this evidence, we describe a significant association between rs12336898 and hypertension in a community-based Korean cohort study. Furthermore, rs12336898 was related to the incidence of hypertension in the baseline low-renin group. Generally, new-onset hypertension increases if renin increases. However, low-renin activity can be an independent predictor of hypertension in Korean individuals [8]. A high level of sodium intake can decrease renin activity due to an increase in blood pressure [43]. Most patients with low-renin hypertension have sodium-volume-dependent hypertension, suggesting that high sodium uptake leads to fluid retention and decreased renin activity to compensate [61,62,63].

In the current study, rs1938859 in the transient receptor potential channel C6 (*TRPC6*) gene was also associated with hypertension. Polymorphisms in the upstream region of the *TRPC6* gene could also result in new-onset hypertension through the RAS. The glomerular filtration barrier consists of podocytes, linked by the slit diaphragm, glomerular basement membrane, and endothelial cells. The slit diaphragm provides physical linkage to the podocytes by connecting podocyte foot processes [64,65]. Mutations in *TRPC6* cause hereditary focal segmental glomerulosclerosis (FSGS), enhancing podocyte expression of wild-type [66,67]. *TRPC6* is related to the slit diaphragm proteins nephrin and podocin, suggesting that *TRPC6* signals the slit diaphragm [68]. Glomerular *TRPC6* expression is elevated in proteinuric diseases, such as FSGS and membranous glomerulopathy [66]. Several *TRPC6* mutations have been identified in the *TRPC6*-encoding gene [69,70].

*TRPC6* is a receptor-operated channel activated by angiotensin II [71,72]. Angiotensin II is an essential contributor to the pathogenesis of the glomerular disease, and it is well known that ACE inhibition and angiotensin type 1 receptor (AT1R) block have antiproteinuric effects [73,74]. In nonrenal cells, Angiotensin II can activate TRPC6 [75,76]. Podocytes in the glomerulus can express both AT1R and AT2R, and Angiotensin II has detrimental effects on podocytes [77,78]. Therefore, *TRPC6* influences podocytes in the glomerulus via angiotensin. When AT1R is overexpressed in podocytes, podocyte damage and glomerulosclerosis are induced in a rat model [79]. Furthermore, overexpression of renin in mice leads to podocyte damage and proteinuria. Such detrimental effects can be improved by treating experimental animals with angiotensin receptor blockers [80].

Some strengths and limitations require careful consideration and may affect the interpretation of the results of the present study. A major strength was that this study was conducted in a large population-based cohort. Furthermore, our findings are drawn from two replicate sample cohorts to validate the associations between relevant SNPs and hypertension. Replication is the gold standard for decreasing false-positive errors in genotype studies [81]. This study had some limitations that should also be acknowledged. First, we did not consider the effects of changes in renin level or the incidence of chronic diseases, such as hepatitis and cancer, which could affect the incidence of hypertension during the follow-up period. Second, the baseline study in KoGES did not contain information on diet, stress, physical activity in detail, which can affect new-onset hypertension. Comprehensively, the interactions between genes, epigenetics, and the environment are complex and influence gene expression [82,83]. Moreover, the data did not include information on which type of antihypertensive medications had been taken. The medications, such as angiotensin II receptor blocker and angiotensin converting enzyme inhibitor, can affect genetic polymorphism, depending on renin levels. Further prospective studies, including changes in renin level and other comorbidities, which can affect the incident hypertension, are needed to demonstrate the genetic polymorphisms as a predictive marker of hypertension more clearly.

## 5. Conclusions

In summary, we revealed an association between rs12336898 in *SPTAN1* and hypertension in the low-renin group. Low-renin individuals could be at high risk of hypertension among Koreans, due to the genetic predisposition with high sodium uptake. Our results might be a valuable indicator for hypertension risk prediction and preventive measures, considering renin levels with genetic susceptibility.

## Figures and Tables

**Table 1:** Baseline characteristics of the study population.

	Low Renin Group			High Renin Group		
	Normotensive	New-Onset Hypertension	*p*-Value	Normotensive	New-Onset Hypertension	*p*-Value
No. subjects (%)	2372	1294		1135	410	
Age (years)	49.2 ± 7.9	53.9 ± 8.8	<0.001	49.1 ± 8.2	51.9 ± 8.8	<0.001
Sex, *n* (%)			0.069			0.009
Male	944 (39.8)	555 (42.9)		667 (58.8)	271 (66.1)	
Female	1428 (60.2)	739 (57.1)		468 (41.2)	139 (33.9)	
Body mass index (kg/m^2^)	24.0 ± 2.9	24.9 ± 3.0	<0.001	23.8 ± 2.9	24.5 ± 3.2	<0.001
Fasting plasma glucose (mg/dL)	88.9 ± 16.6	91.8 ± 20.7	<0.001	92.2 ± 23.1	96.5 ± 35.0	0.007
Total cholesterol (mg/dL)	193.0 ± 34.7	196.1 ± 34.8	0.016	199.6 ± 34.5	202.4 ± 38.2	0.173
Triglycerides (mg/dL)	132.4 ± 92.0	150.9 ± 99.7	<0.001	141.1 ± 97.9	168.9 ± 117.8	<0.001
HDL cholesterol (mg/dL)	50.2 ± 11.7	48.4 ± 11.2	<0.001	50.3 ± 11.9	49.7 ± 12.2	0.379
Systolic blood pressure (mmHg)	120.8 ± 19.7	121.1 ± 19.3	0.663	119.8 ± 18.0	120.6 ± 18.9	0.449
Diastolic blood pressure (mmHg)	88.9 ± 16.6	79.9 ± 12.4	0.337	79.8 ± 11.4	79.3 ± 11.9	0.450
Current smoker, *n* (%)	509 (21.4)	323 (24.9)	0.015	361 (31.8)	143 (34.9)	0.256
Diabetes, *n* (%)	165 (7.0)	167 (13.0)	<0.001	103 (9.1)	64 (15.6)	<0.001

Data are presented as mean ± SD, proportion, or median (interquartile range) for skewed variables. *p*-values were calculated by one-way ANOVA for continuous variables and chi-square test for categorical variables.

**Table 2:** Top significant SNP of three hypertension case-control GWASs in the association cluster locus.

CHR	SNP	BP	A1	Total Hypertension Cases (*n*= 1704) and Controls (*n* = 3507)				Low Renin Group Cases (*n* = 1294) and Controls (*n* = 2372)				High Renin Group Cases (*n* = 410) and Controls (*n* = 1135)				Associations by Renin Group	Locus Top	Cluster Pick
				OR	L95	U95	*p*	OR	L95	U95	P	OR	L95	U95	*p*			
4	rs11726091	4,952,412	C	1.14	1.04	1.24	3.3 × 10^−3^	1.03	0.93	1.14	5.5 × 10^−1^	1.48	1.25	1.75	4.2 × 10^−6^	H	T	C
4	rs17038966	109,296,214	A	0.72	0.60	0.86	2.1 × 10^−4^	0.62	0.50	0.76	6.0 × 10^−6^	1.05	0.76	1.44	7.7 × 10^−1^	L	T	C
4	rs145286444	167,264,137	A	1.33	1.15	1.55	2.1 × 10^−4^	1.54	1.28	1.84	2.9 × 10^−6^	0.97	0.72	1.30	8.2 × 10^−1^	L	T	C
5	rs2118663	153,340,145	C	1.26	1.10	1.44	1.0 × 10^−3^	1.46	1.24	1.71	3.8 × 10^−6^	0.81	0.61	1.08	1.5 × 10^−1^	L	T	C
9	rs12336898	131,305,177	T	0.85	0.78	0.93	4.8 × 10^−4^	0.77	0.69	0.86	1.3 × 10^−6^	1.09	0.93	1.29	2.9 × 10^−1^	L	T	C
11	rs1938859	101,547,723	T	1.38	1.20	1.58	4.6 × 10^−6^	1.40	1.19	1.64	4.7 × 10^−5^	1.35	1.04	1.75	2.3 × 10^−2^	M	T	C
12	rs7968218	94,189,185	C	0.74	0.65	0.84	4.1 × 10^−6^	0.75	0.65	0.88	2.3 × 10^−4^	0.68	0.53	0.88	3.9 × 10^−3^	M	T	C
18	rs117246401	32,975,483	T	1.71	1.37	2.14	1.8 × 10^−6^	1.57	1.21	2.04	6.6 × 10^−4^	2.09	1.38	3.16	5.1 × 10^−4^	M	T	C
22	rs8137145	25,213,480	T	0.83	0.76	0.91	6.6 × 10^−5^	0.91	0.82	1.01	7.7 × 10^−2^	0.67	0.57	0.80	6.4 × 10^−6^	H	T	C

CHR, chromosome; SNP, single nucleotide polymorphism; BP, base pair; A1, minor allele; OR, odds ratio; L95, lower 95% confidence interval; U95, upper 95% confidence interval; *p*, *p*-value; L, low-renin group; H, high-renin group; M, both groups; T, top significant SNP in the locus; C, cluster more than three SNPs in each locus with *p*-values < 1 × 10^−5^, adjusted for sex, age, and BMI.

**Table 3:** Interpretations of significant SNPs.

CHR	SNP	BP	A1	Renin Group	Locus	Nearby Genes (±100 kbp)	Function	Previous GWAS (±100 kbp)	eQTL
4	rs11726091	4,952,412	C	H	4p16.2	MSX1 CYTL1	Intergenic	Body mass index, waist-hip ratio, post bronchodilator FEV1/FVC ratio, lung function (FVC), pediatric autoimmune diseases [17,18,19]	*MSX1* (artery-aorta, muscle-skeletal)
4	rs17038966	109,296,214	A	L	4q25	No gene	Intergenic	Lung function (FVC), hair colour, tooth agenesis, airflow obstruction [20,21,22]	
4	rs145286444	167,264,137	A	L	4q32.3	TTL1	Intergenic	Velopharyngeal dysfunction, serous borderline ovarian cancer, obesity-related traits [23,24,25]	
5	rs2118663	153,340,145	C	L	5q33.2	FAM114A2 MFAP3	3′ downstream	Educational attainment (MTAG), red cell distribution width, multiple sclerosis [26,27,28]	*FAM114A2* (muscle-skeletal, whole-blood, heart-atrial appendage, brain-frontal-cortex, adipose-subcutaneous, cultured-fibroblasts)
9	rs12336898	131,305,177	T	L	9q34.11	ODF2 GLE1 SPTAN1	3′ downstream	Axial length, pulse pressure [29]	
11	rs1938859	101,547,723	T	M	11q22.1	TRPC6	5′ upstream	Sleep duration, general risk tolerance (MTAG), change in LVEF in response to paclitaxel and trastuzumab in HER2+ breast cancer [30,31,32]	*TRPC6* (colon-sigmoid, pancreas, heart-left-ventricle, nerve-tibial)
12	rs7968218	94,189,185	C	M	12q22	CRADD	Intron	Lung function (FVC), waist-to-hip ratio, Alzheimer’s disease (cognitive decline) [33,34,35]	
18	rs117246401	32,975,483	T	M	18q12.2	ZNF271P, ZNF396, INO80C	Intergenic	Highest math class taken (MTAG), Parkinson’s disease, well-being spectrum (multivariate analysis), neuroticism, life satisfaction, parasitaemia in Tripanosoma cruzi seropositivity [36,37,38]	
22	rs8137145	25,213,480	T	H	22q11.23	PIWIL3 SGSM1	Intron of SGSM1	Macrophage migration inhibitory factor levels, nicotine dependence and major depression (severity of comorbidity), resting-state electroencephalogram vigilance [39,40,41]	*SGSM1* (muscle-skeletal, lung, adipose-visceral, spleen, pancreas, whole-blood)

## Data Availability

Data in this study were from the Korean Genome and Epidemiology Study (KoGES; 4851-302). These data are available through an online sharing service, under the permission of the division of epidemiology and health index in the Korea Centers for Disease Control and Prevention (KCDC), at http://www.nih.go.kr/NIH/eng/contents/ (accessed on 14 March 2022).

## Supplementary Materials

The following supporting information can be downloaded at: https://www.mdpi.com/article/10.3390/jcdd9040104/s1, Table S1: Series of SNPs for association analysis of hypertension and renin-related SNPs.
