# Supplementary material for: Genome-Wide Association of New-Onset Hypertension According to Renin Concentration: The Korean Genome and Epidemiology Cohort Study
Source: J Cardiovasc Dev Dis. 2022 Mar 30;9(4):104. doi: 10.3390/jcdd9040104 (PMC9025963; doi:10.3390/jcdd9040104)
Supplement: Supplementary file 1 [file jcdd-09-00104-s001.zip › jcdd-1612544-supplementary.pdf]

Supplementary

Table S1. Series of SNPs for association analysis of hypertension and renin-related SNPs

CHR	SNP	BP	A1	Total hypertension new onset hypertension (n= 1704) and Normotensive (n=3507)					Low renin group new onset hypertension (n=1294) and Normotensive (n=2372)					High renin group new onset hypertension (n=410) and Normotensive (n=1135)				
				NMISS	OR	L95	U95	P	NMISS	OR	L95	U95	P	NMISS	OR	L95	U95	P
2	rs7599476	186280606	A	5211	1.16	1.06	1.26	7.2E-04	3666	1.26	1.14	1.39	6.8E-06	1545	0.93	0.79	1.10	3.9E-01
2	rs10177733	186283241	T	5211	1.16	1.06	1.26	7.0E-04	3666	1.26	1.14	1.39	6.8E-06	1545	0.93	0.79	1.10	4.0E-01
2	rs80257808	201100320	C	5211	1.39	1.16	1.65	2.8E-04	3666	1.67	1.35	2.06	2.0E-06	1545	0.89	0.63	1.25	4.9E-01
3	3:143886786:AT_A	143886786	D	5211	1.34	1.14	1.57	3.4E-04	3666	1.56	1.30	1.88	2.6E-06	1545	0.82	0.59	1.15	2.5E-01
3	rs141061779	162494104	C	5211	0.64	0.52	0.78	1.3E-05	3666	0.58	0.46	0.73	5.2E-06	1545	0.78	0.53	1.17	2.3E-01
4	rs11725871	4951819	A	5211	1.14	1.05	1.25	2.8E-03	3666	1.04	0.93	1.15	5.1E-01	1545	1.48	1.25	1.75	4.4E-06
4	rs11726091	4952412	C	5211	1.14	1.04	1.24	3.3E-03	3666	1.03	0.93	1.14	5.5E-01	1545	1.48	1.25	1.75	4.2E-06
4	rs4689189	4952895	T	5211	1.14	1.04	1.24	4.3E-03	3666	1.03	0.93	1.14	5.8E-01	1545	1.47	1.24	1.74	6.3E-06
4	rs2968709	4954275	C	5211	1.14	1.04	1.24	4.4E-03	3666	1.03	0.93	1.14	5.8E-01	1545	1.47	1.24	1.74	6.4E-06
4	rs4689191	4962911	T	5211	1.14	1.05	1.25	3.0E-03	3666	1.04	0.93	1.15	5.2E-01	1545	1.48	1.25	1.75	4.4E-06
4	rs17038966	109296214	A	5211	0.72	0.60	0.86	2.1E-04	3666	0.62	0.50	0.76	6.0E-06	1545	1.05	0.76	1.44	7.7E-01
4	rs17038968	109296717	C	5211	0.72	0.60	0.86	2.1E-04	3666	0.62	0.50	0.76	6.0E-06	1545	1.05	0.76	1.44	7.7E-01
4	rs740053	109299202	A	5211	0.72	0.60	0.85	1.8E-04	3666	0.62	0.51	0.77	7.7E-06	1545	1.02	0.74	1.40	9.0E-01
4	rs77619314	109299707	A	5211	0.72	0.60	0.85	1.8E-04	3666	0.62	0.51	0.77	7.7E-06	1545	1.02	0.74	1.40	9.0E-01
4	rs17551860	111418814	A	5211	1.19	1.05	1.34	5.6E-03	3666	1.03	0.89	1.20	6.9E-01	1545	1.67	1.34	2.08	4.8E-06
4	rs1460063	166804183	G	5211	1.07	0.96	1.19	2.1E-01	3666	0.92	0.82	1.04	2.0E-01	1545	1.57	1.29	1.92	9.7E-06
4	rs2125862	166806601	A	5211	1.06	0.96	1.18	2.7E-01	3666	0.91	0.81	1.03	1.4E-01	1545	1.57	1.29	1.92	9.6E-06
4	rs79847934	167190224	A	5211	1.33	1.14	1.55	2.0E-04	3666	1.50	1.26	1.80	7.6E-06	1545	1.01	0.75	1.36	9.3E-01
4	rs353077	167259818	A	5211	1.30	1.12	1.51	5.0E-04	3666	1.51	1.26	1.80	5.1E-06	1545	0.95	0.71	1.27	7.1E-01
4	rs9992643	167260185	T	5211	1.30	1.12	1.51	6.3E-04	3666	1.51	1.27	1.81	4.9E-06	1545	0.93	0.70	1.25	6.3E-01
4	rs10027143	167260264	C	5211	1.30	1.12	1.51	5.2E-04	3666	1.52	1.27	1.81	4.0E-06	1545	0.93	0.70	1.25	6.3E-01
4	rs73861252	167263637	C	5211	1.30	1.12	1.51	5.0E-04	3666	1.51	1.27	1.81	4.7E-06	1545	0.94	0.70	1.26	6.8E-01
4	rs145286444	167264137	A	5211	1.33	1.15	1.55	2.1E-04	3666	1.54	1.28	1.84	2.9E-06	1545	0.97	0.72	1.30	8.2E-01
4	rs73861253	167264848	A	5211	1.30	1.12	1.51	5.0E-04	3666	1.51	1.27	1.81	4.7E-06	1545	0.94	0.70	1.26	6.8E-01
5	rs118133352	62134036	G	5211	2.37	1.70	3.31	3.9E-07	3666	2.64	1.72	4.03	7.8E-06	1545	2.17	1.26	3.73	5.4E-03
5	rs1025260	153335137	T	5211	1.24	1.09	1.42	1.6E-03	3666	1.44	1.23	1.68	5.5E-06	1545	0.81	0.61	1.06	1.3E-01
5	rs6883500	153338535	T	5211	1.24	1.09	1.42	1.6E-03	3666	1.44	1.23	1.68	5.5E-06	1545	0.81	0.61	1.06	1.3E-01
5	rs2118662	153340134	T	5211	1.24	1.09	1.42	1.6E-03	3666	1.44	1.23	1.68	5.5E-06	1545	0.81	0.61	1.06	1.3E-01
5	rs2118663	153340145	C	5211	1.26	1.10	1.44	1.0E-03	3666	1.46	1.24	1.71	3.8E-06	1545	0.81	0.61	1.08	1.5E-01
5	rs72804698	153342954	A	5211	1.24	1.08	1.41	1.9E-03	3666	1.43	1.22	1.68	7.3E-06	1545	0.81	0.61	1.06	1.3E-01
5	rs66607728	153343118	G	5211	1.23	1.08	1.41	1.9E-03	3666	1.43	1.22	1.67	6.7E-06	1545	0.80	0.61	1.05	1.1E-01
5	rs7710732	153443533	G	5211	1.25	1.10	1.41	6.6E-04	3666	1.40	1.21	1.63	7.4E-06	1545	0.88	0.68	1.13	3.2E-01
5	rs4081999	153452773	A	5211	1.25	1.10	1.42	5.2E-04	3666	1.41	1.21	1.63	6.5E-06	1545	0.89	0.69	1.14	3.5E-01
5	rs34195048	153474851	T	5211	1.24	1.10	1.41	7.5E-04	3666	1.40	1.21	1.62	6.9E-06	1545	0.87	0.67	1.12	2.7E-01
5	5:153475405:TG_T	153475405	D	5211	1.24	1.09	1.41	7.9E-04	3666	1.40	1.21	1.62	6.9E-06	1545	0.86	0.67	1.11	2.6E-01
5	rs4312933	153476659	T	5211	1.24	1.10	1.41	7.1E-04	3666	1.40	1.21	1.63	6.1E-06	1545	0.86	0.67	1.11	2.6E-01
5	rs2882468	153476822	T	5211	1.24	1.10	1.41	7.1E-04	3666	1.40	1.21	1.63	6.1E-06	1545	0.86	0.67	1.11	2.6E-01
5	rs6580052	153478177	C	5211	1.24	1.10	1.41	6.6E-04	3666	1.40	1.21	1.63	6.1E-06	1545	0.87	0.67	1.12	2.7E-01

5	rs2434375	153479613	G	5211	1.24	1.10	1.41	7.4E-04	3666	1.40	1.21	1.63	6.1E-06	1545	0.86	0.67	1.11	2.5E-01
5	rs815638	153480265	G	5211	1.24	1.09	1.40	8.2E-04	3666	1.40	1.21	1.62	7.0E-06	1545	0.86	0.67	1.11	2.5E-01
6	rs12200265	76814691	A	5211	0.67	0.56	0.79	5.8E-06	3666	0.64	0.52	0.79	3.0E-05	1545	0.75	0.54	1.04	7.9E-02
6	rs9341551	76821206	A	5211	0.67	0.56	0.80	8.8E-06	3666	0.63	0.50	0.78	2.3E-05	1545	0.79	0.57	1.10	1.6E-01
7	7:9855688:T_TAG	9855688	I	5211	0.77	0.64	0.93	6.1E-03	3666	0.99	0.80	1.23	9.6E-01	1545	0.39	0.26	0.59	7.7E-06
7	rs150955441	23622861	G	5211	1.37	1.21	1.56	1.7E-06	3666	1.43	1.22	1.66	6.1E-06	1545	1.26	0.99	1.62	6.5E-02
8	rs117963869	77498540	T	5211	1.18	1.06	1.31	2.2E-03	3666	1.05	0.93	1.19	4.2E-01	1545	1.55	1.28	1.87	8.1E-06
9	rs10968821	2899925	G	5211	0.83	0.75	0.91	1.5E-04	3666	0.76	0.68	0.86	6.5E-06	1545	1.01	0.84	1.22	9.0E-01
9	rs2376415	2904934	C	5211	0.83	0.75	0.91	1.1E-04	3666	0.76	0.67	0.85	2.2E-06	1545	1.02	0.85	1.22	8.3E-01
9	rs9284028	16375458	G	5211	0.82	0.75	0.89	7.0E-06	3666	0.85	0.76	0.94	1.2E-03	1545	0.75	0.63	0.88	5.9E-04
9	rs4327921	74750736	A	5211	0.80	0.71	0.90	1.9E-04	3666	0.72	0.63	0.83	7.1E-06	1545	1.04	0.84	1.30	7.1E-01
9	rs4836545	129251213	G	5211	0.84	0.77	0.92	8.2E-05	3666	0.79	0.71	0.87	5.1E-06	1545	0.99	0.84	1.17	8.9E-01
9	rs10987302	129253264	G	5211	0.84	0.77	0.92	1.2E-04	3666	0.78	0.70	0.87	2.8E-06	1545	1.03	0.87	1.21	7.6E-01
9	9:129253699:ATGT_	129253699	D	5211	0.84	0.77	0.92	1.2E-04	3666	0.78	0.70	0.87	2.6E-06	1545	1.03	0.87	1.21	7.6E-01
9	rs4837266	131205954	C	5211	0.86	0.79	0.94	1.1E-03	3666	0.79	0.71	0.87	6.9E-06	1545	1.09	0.92	1.29	3.4E-01
9	rs10987989	131206510	G	5211	0.86	0.79	0.94	1.1E-03	3666	0.79	0.71	0.87	7.2E-06	1545	1.09	0.92	1.29	3.3E-01
9	rs55649012	131206797	G	5211	0.86	0.79	0.94	9.6E-04	3666	0.78	0.71	0.87	6.0E-06	1545	1.08	0.92	1.28	3.5E-01
9	rs7866034	131209484	T	5211	0.87	0.79	0.95	1.8E-03	3666	0.78	0.70	0.87	5.3E-06	1545	1.12	0.95	1.32	1.9E-01
9	rs112793868	131209653	A	5211	0.87	0.79	0.95	1.6E-03	3666	0.78	0.70	0.87	5.2E-06	1545	1.11	0.94	1.32	2.1E-01
9	rs58657174	131210026	G	5211	0.87	0.79	0.95	1.7E-03	3666	0.78	0.70	0.87	5.0E-06	1545	1.12	0.94	1.32	2.0E-01
9	rs12377955	131210080	A	5211	0.86	0.79	0.94	1.1E-03	3666	0.78	0.70	0.86	2.9E-06	1545	1.11	0.94	1.31	2.2E-01
9	rs7866619	131210115	T	5211	0.87	0.79	0.95	1.7E-03	3666	0.78	0.70	0.87	4.9E-06	1545	1.12	0.94	1.32	2.0E-01
9	rs7867073	131210328	A	5211	0.87	0.79	0.95	1.6E-03	3666	0.78	0.70	0.87	4.6E-06	1545	1.11	0.94	1.32	2.1E-01
9	rs7869756	131210410	G	5211	0.87	0.79	0.95	1.9E-03	3666	0.79	0.71	0.87	7.4E-06	1545	1.11	0.94	1.31	2.3E-01
9	rs7870167	131210643	T	5211	0.87	0.79	0.95	1.6E-03	3666	0.78	0.70	0.87	4.6E-06	1545	1.11	0.94	1.32	2.1E-01
9	rs75928353	131210823	G	5211	0.87	0.79	0.95	1.6E-03	3666	0.78	0.70	0.87	4.6E-06	1545	1.12	0.94	1.32	2.0E-01
9	rs7874255	131211537	T	5211	0.87	0.79	0.95	1.5E-03	3666	0.78	0.70	0.87	4.0E-06	1545	1.12	0.95	1.32	1.9E-01
9	rs4836612	131211691	G	5211	0.86	0.79	0.94	1.2E-03	3666	0.78	0.70	0.87	4.6E-06	1545	1.10	0.93	1.30	2.6E-01
9	9:131211827:TCAG_	131211827	D	5211	0.86	0.79	0.94	1.3E-03	3666	0.78	0.70	0.87	4.2E-06	1545	1.11	0.93	1.31	2.4E-01
9	9:131211828:CAGG_	131211828	D	5211	0.87	0.79	0.95	1.6E-03	3666	0.78	0.70	0.87	4.3E-06	1545	1.12	0.94	1.32	2.0E-01
9	rs10121184	131211934	G	5211	0.87	0.79	0.95	1.9E-03	3666	0.79	0.71	0.87	7.4E-06	1545	1.11	0.94	1.32	2.2E-01
9	rs7874633	131211953	G	5211	0.87	0.79	0.95	1.5E-03	3666	0.78	0.70	0.87	3.7E-06	1545	1.12	0.95	1.32	1.9E-01
9	rs10115137	131212105	T	5211	0.87	0.79	0.95	1.5E-03	3666	0.78	0.70	0.87	4.0E-06	1545	1.12	0.95	1.32	1.9E-01
9	rs7849186	131212372	G	5211	0.86	0.79	0.94	9.9E-04	3666	0.78	0.70	0.87	3.4E-06	1545	1.10	0.93	1.30	2.6E-01
9	9:131212504:T_TTT	131212504	I	5211	0.87	0.79	0.95	1.5E-03	3666	0.78	0.70	0.87	5.1E-06	1545	1.11	0.94	1.31	2.3E-01
9	rs10117399	131212766	G	5211	0.87	0.79	0.95	1.5E-03	3666	0.78	0.70	0.87	3.7E-06	1545	1.12	0.95	1.32	1.9E-01
9	rs7849384	131212831	C	5211	0.87	0.79	0.95	1.6E-03	3666	0.78	0.70	0.87	4.1E-06	1545	1.12	0.95	1.32	1.9E-01
9	rs10124474	131213984	T	5211	0.87	0.79	0.95	1.5E-03	3666	0.78	0.70	0.87	4.0E-06	1545	1.12	0.95	1.32	1.9E-01
9	rs10124529	131214254	T	5211	0.87	0.79	0.95	1.5E-03	3666	0.78	0.70	0.87	4.0E-06	1545	1.12	0.95	1.32	1.9E-01
9	rs4240429	131219877	C	5211	0.86	0.79	0.94	9.6E-04	3666	0.78	0.70	0.87	3.2E-06	1545	1.10	0.93	1.30	2.6E-01
9	rs10987999	131226457	T	5211	0.86	0.79	0.94	9.6E-04	3666	0.78	0.70	0.87	3.2E-06	1545	1.10	0.93	1.30	2.6E-01
9	rs10988000	131228566	A	5211	0.86	0.79	0.94	9.6E-04	3666	0.78	0.70	0.87	3.2E-06	1545	1.10	0.93	1.30	2.6E-01
9	rs73626757	131229372	C	5211	0.86	0.79	0.94	9.6E-04	3666	0.78	0.70	0.87	3.2E-06	1545	1.10	0.93	1.30	2.6E-01
9	9:131237610:CTG_C	131237610	D	5211	0.86	0.79	0.94	9.0E-04	3666	0.78	0.70	0.87	3.3E-06	1545	1.10	0.93	1.30	2.8E-01
9	rs28705141	131238797	G	5211	0.86	0.79	0.94	9.6E-04	3666	0.78	0.70	0.87	3.2E-06	1545	1.10	0.93	1.30	2.6E-01
9	rs4837271	131241808	G	5211	0.86	0.79	0.94	9.9E-04	3666	0.78	0.70	0.86	3.0E-06	1545	1.10	0.93	1.30	2.4E-01
9	9:131241924:CT_C	131241924	D	5211	0.86	0.79	0.94	1.0E-03	3666	0.78	0.70	0.86	3.0E-06	1545	1.11	0.94	1.31	2.4E-01
9	rs3924786	131247626	A	5211	0.86	0.79	0.94	9.9E-04	3666	0.78	0.70	0.86	3.0E-06	1545	1.10	0.93	1.30	2.4E-01

9	rs4268218	131248122	A	5211	0.86	0.79	0.94	9.9E-04	3666	0.78	0.70	0.86	3.0E-06	1545	1.10	0.93	1.30	2.4E-01
9	rs4837272	131248748	G	5211	0.86	0.79	0.94	1.1E-03	3666	0.78	0.70	0.87	3.2E-06	1545	1.11	0.94	1.31	2.4E-01
9	rs4837273	131249045	G	5211	0.86	0.79	0.94	9.9E-04	3666	0.78	0.70	0.86	3.0E-06	1545	1.10	0.93	1.30	2.4E-01
9	rs111396465	131249488	G	5211	0.86	0.79	0.94	8.4E-04	3666	0.78	0.70	0.87	3.2E-06	1545	1.09	0.93	1.29	2.9E-01
9	rs7847306	131254439	T	5211	0.86	0.79	0.94	9.9E-04	3666	0.78	0.70	0.86	3.0E-06	1545	1.10	0.93	1.30	2.4E-01
9	rs10819390	131256166	T	5211	0.86	0.79	0.94	9.9E-04	3666	0.78	0.70	0.86	3.0E-06	1545	1.10	0.93	1.30	2.4E-01
9	rs4837274	131257202	G	5211	0.86	0.79	0.94	9.9E-04	3666	0.78	0.70	0.86	3.0E-06	1545	1.10	0.93	1.30	2.4E-01
9	rs10988009	131261864	C	5211	0.86	0.79	0.94	1.0E-03	3666	0.78	0.70	0.86	3.0E-06	1545	1.11	0.94	1.31	2.4E-01
9	rs28868410	131270256	G	5211	0.86	0.79	0.94	6.8E-04	3666	0.77	0.70	0.86	1.8E-06	1545	1.10	0.93	1.30	2.6E-01
9	rs4614100	131270480	T	5211	0.86	0.79	0.94	6.8E-04	3666	0.77	0.70	0.86	1.8E-06	1545	1.10	0.93	1.30	2.6E-01
9	rs7034108	131275853	G	5211	0.86	0.79	0.94	6.4E-04	3666	0.77	0.70	0.86	1.8E-06	1545	1.10	0.93	1.30	2.7E-01
9	rs78557279	131283161	A	5211	0.86	0.79	0.94	6.4E-04	3666	0.77	0.70	0.86	1.8E-06	1545	1.10	0.93	1.30	2.7E-01
9	rs13439919	131287118	G	5211	0.86	0.79	0.94	6.4E-04	3666	0.77	0.70	0.86	1.8E-06	1545	1.10	0.93	1.30	2.7E-01
9	rs10988015	131287956	T	5211	0.86	0.79	0.94	6.4E-04	3666	0.77	0.70	0.86	1.8E-06	1545	1.10	0.93	1.30	2.7E-01
9	rs28361547	131288441	T	5211	0.86	0.79	0.94	6.4E-04	3666	0.77	0.70	0.86	1.8E-06	1545	1.10	0.93	1.30	2.7E-01
9	rs869455	131289600	T	5211	0.86	0.79	0.94	6.4E-04	3666	0.77	0.70	0.86	1.8E-06	1545	1.10	0.93	1.30	2.7E-01
9	rs12339729	131294316	G	5211	0.86	0.79	0.94	6.8E-04	3666	0.77	0.70	0.86	1.8E-06	1545	1.10	0.93	1.30	2.6E-01
9	rs10988021	131296580	A	5211	0.86	0.79	0.94	6.8E-04	3666	0.77	0.70	0.86	1.8E-06	1545	1.10	0.93	1.30	2.6E-01
9	rs4836614	131301651	C	5211	0.86	0.79	0.94	6.5E-04	3666	0.77	0.70	0.86	1.7E-06	1545	1.10	0.93	1.30	2.6E-01
9	rs10760563	131303522	A	5211	0.86	0.79	0.94	6.7E-04	3666	0.77	0.70	0.86	1.8E-06	1545	1.10	0.93	1.30	2.6E-01
9	rs12336898	131305177	T	5211	0.85	0.78	0.93	4.8E-04	3666	0.77	0.69	0.86	1.3E-06	1545	1.09	0.93	1.29	2.9E-01
9	rs10988033	131306479	G	5211	0.86	0.79	0.94	6.5E-04	3666	0.77	0.70	0.86	1.7E-06	1545	1.10	0.93	1.30	2.6E-01
9	rs10819398	131309047	A	5211	0.86	0.79	0.94	6.5E-04	3666	0.77	0.70	0.86	1.7E-06	1545	1.10	0.93	1.30	2.6E-01
9	rs10819399	131309059	A	5211	0.86	0.79	0.94	6.5E-04	3666	0.77	0.70	0.86	1.7E-06	1545	1.10	0.93	1.30	2.6E-01
9	rs10819400	131309147	G	5211	0.86	0.79	0.94	6.5E-04	3666	0.77	0.70	0.86	1.7E-06	1545	1.10	0.93	1.30	2.6E-01
9	rs12236739	131309995	G	5211	0.86	0.79	0.94	6.5E-04	3666	0.77	0.70	0.86	1.7E-06	1545	1.10	0.93	1.30	2.6E-01
9	rs10513497	131311128	C	5211	0.86	0.79	0.94	6.5E-04	3666	0.77	0.70	0.86	1.7E-06	1545	1.10	0.93	1.30	2.6E-01
9	rs11999274	131311333	A	5211	0.86	0.79	0.94	6.5E-04	3666	0.77	0.70	0.86	1.7E-06	1545	1.10	0.93	1.30	2.6E-01
10	rs10870235	133879820	G	5211	0.80	0.72	0.89	2.2E-05	3666	0.76	0.67	0.85	7.7E-06	1545	0.91	0.75	1.11	3.6E-01
11	rs1938859	101547723	T	5211	1.38	1.20	1.58	4.6E-06	3666	1.40	1.19	1.64	4.7E-05	1545	1.35	1.04	1.75	2.3E-02
11	rs4536179	101555121	T	5211	1.37	1.20	1.57	5.9E-06	3666	1.38	1.18	1.62	8.2E-05	1545	1.37	1.06	1.78	1.8E-02
11	rs62951762	101556143	G	5211	1.37	1.19	1.57	7.7E-06	3666	1.38	1.18	1.62	8.2E-05	1545	1.35	1.04	1.76	2.4E-02
11	rs11224957	101557676	C	5211	1.37	1.19	1.57	6.7E-06	3666	1.38	1.18	1.62	8.2E-05	1545	1.36	1.05	1.77	2.0E-02
11	rs12786238	101558792	A	5211	1.37	1.19	1.57	6.7E-06	3666	1.38	1.18	1.62	8.2E-05	1545	1.36	1.05	1.77	2.0E-02
11	rs11224962	101562330	A	5211	1.37	1.20	1.57	5.0E-06	3666	1.39	1.19	1.64	5.1E-05	1545	1.35	1.04	1.75	2.3E-02
11	rs11224968	101564618	T	5211	1.37	1.20	1.57	5.0E-06	3666	1.39	1.19	1.64	5.1E-05	1545	1.35	1.04	1.75	2.3E-02
12	rs7964898	51498552	C	5211	1.99	1.48	2.67	4.9E-06	3666	2.02	1.43	2.86	7.0E-05	1545	1.83	1.04	3.23	3.6E-02
12	rs2730660	51524025	T	5211	1.26	1.13	1.40	4.9E-05	3666	1.35	1.18	1.53	7.7E-06	1545	1.03	0.84	1.28	7.6E-01
12	rs148621332	59405696	C	5211	1.28	0.91	1.80	1.6E-01	3666	0.78	0.51	1.18	2.4E-01	1545	4.22	2.24	7.95	8.9E-06
12	12:94188182:A_AC	94188182	I	5211	0.74	0.65	0.84	4.5E-06	3666	0.75	0.65	0.88	2.3E-04	1545	0.69	0.53	0.89	4.2E-03
12	rs7968218	94189185	C	5211	0.74	0.65	0.84	4.1E-06	3666	0.75	0.65	0.88	2.3E-04	1545	0.68	0.53	0.88	3.9E-03
12	rs7953679	94189501	G	5211	0.74	0.65	0.84	4.1E-06	3666	0.75	0.65	0.88	2.3E-04	1545	0.68	0.53	0.88	3.9E-03
12	rs11107191	94190902	T	5211	0.74	0.65	0.84	5.0E-06	3666	0.75	0.65	0.88	2.3E-04	1545	0.69	0.53	0.90	5.1E-03
12	rs12578889	94191575	A	5211	0.74	0.65	0.84	4.1E-06	3666	0.75	0.65	0.88	2.3E-04	1545	0.68	0.53	0.88	3.9E-03
12	rs12581702	94192225	G	5211	0.74	0.65	0.84	6.6E-06	3666	0.76	0.65	0.88	3.3E-04	1545	0.69	0.53	0.89	4.8E-03
12	rs12581704	94192252	G	5211	0.74	0.65	0.84	4.1E-06	3666	0.75	0.65	0.88	2.3E-04	1545	0.68	0.53	0.88	3.9E-03
12	rs17021583	94192644	T	5211	0.74	0.65	0.84	4.1E-06	3666	0.75	0.65	0.88	2.3E-04	1545	0.68	0.53	0.88	3.9E-03
12	rs2131724	94194170	C	5211	0.74	0.65	0.84	5.1E-06	3666	0.76	0.65	0.88	2.9E-04	1545	0.68	0.53	0.88	3.9E-03

12	rs59426038	94200026	A	5211	0.74	0.65	0.85	7.6E-06	3666	0.76	0.65	0.88	3.1E-04	1545	0.70	0.54	0.90	5.9E-03
12	rs1493856	94200557	G	5211	0.75	0.66	0.85	7.3E-06	3666	0.78	0.68	0.91	9.9E-04	1545	0.66	0.51	0.84	1.1E-03
12	rs6538669	96132596	G	5211	0.81	0.73	0.90	1.4E-04	3666	0.74	0.65	0.84	5.3E-06	1545	1.02	0.83	1.25	8.6E-01
13	13:69826026:A_ATG	69826026	R	5211	1.27	0.95	1.68	1.0E-01	3666	0.84	0.59	1.22	3.7E-01	1545	2.99	1.85	4.85	8.3E-06
13	rs4545677	76512176	C	5211	1.26	1.00	1.58	4.9E-02	3666	0.92	0.70	1.21	5.6E-01	1545	2.70	1.78	4.11	3.5E-06
14	rs10136621	101017490	T	5211	1.78	1.39	2.29	5.7E-06	3666	1.72	1.27	2.33	4.7E-04	1545	2.00	1.29	3.11	2.0E-03
18	rs143483638	32800139	T	5211	1.73	1.36	2.20	9.0E-06	3666	1.68	1.26	2.23	3.4E-04	1545	1.84	1.15	2.93	1.1E-02
18	rs76835068	32815162	A	5211	1.74	1.36	2.21	7.5E-06	3666	1.65	1.24	2.19	5.8E-04	1545	1.98	1.25	3.13	3.7E-03
18	rs148088942	32941304	C	5211	1.69	1.36	2.10	2.0E-06	3666	1.56	1.20	2.01	7.1E-04	1545	2.06	1.37	3.10	5.6E-04
18	rs117246401	32975483	T	5211	1.71	1.37	2.14	1.8E-06	3666	1.57	1.21	2.04	6.6E-04	1545	2.09	1.38	3.16	5.1E-04
22	rs34190801	25212471	R	5211	0.83	0.76	0.91	4.7E-05	3666	0.90	0.81	1.00	5.8E-02	1545	0.67	0.56	0.80	7.3E-06
22	rs11704456	25212702	G	5211	0.83	0.76	0.91	5.2E-05	3666	0.90	0.81	1.00	5.8E-02	1545	0.67	0.57	0.80	9.4E-06
22	rs67278363	25212863	T	5211	0.83	0.76	0.91	5.9E-05	3666	0.90	0.81	1.01	6.2E-02	1545	0.67	0.57	0.80	9.4E-06
22	rs16978992	25213023	C	5211	0.83	0.76	0.91	5.1E-05	3666	0.90	0.81	1.00	6.0E-02	1545	0.67	0.57	0.80	8.6E-06
22	rs9680840	25213443	C	5211	0.84	0.77	0.92	1.2E-04	3666	0.92	0.82	1.02	1.0E-01	1545	0.68	0.57	0.80	9.1E-06
22	rs9680841	25213476	C	5211	0.84	0.77	0.92	1.2E-04	3666	0.92	0.83	1.02	1.1E-01	1545	0.67	0.57	0.80	7.2E-06
22	rs8137145	25213480	T	5211	0.83	0.76	0.91	6.6E-05	3666	0.91	0.82	1.01	7.7E-02	1545	0.67	0.57	0.80	6.4E-06
22	rs11360399	25213669	R	5211	0.83	0.76	0.91	4.6E-05	3666	0.90	0.81	1.00	5.4E-02	1545	0.68	0.57	0.80	9.7E-06
22	22:25213674:AG_A	25213674	R	5211	0.83	0.76	0.91	5.0E-05	3666	0.90	0.81	1.00	5.7E-02	1545	0.68	0.57	0.80	9.7E-06
